# The influence of race duration on oxygen demand, uptake and deficit in competitive cross-country skiers

**DOI:** 10.1007/s00421-024-05531-1

**Published:** 2024-06-25

**Authors:** Øyvind Gløersen, Ånung Viken, Magne Lund-Hansen, Thomas Losnegard

**Affiliations:** 1 Department of Health Research, SINTEF Digital, Postboks 124 Blindern, 0314 Oslo, Norway; 2 Department of Physical Performance, Norwegian School of Sport Sciences, Oslo, Norway

**Keywords:** Metabolic rate, Oxygen consumption, Anaerobic capacity, Oxygen deficit, Power duration, Intermittent exercise

## Abstract

**Purpose:**

To measure oxygen demand, uptake, and deficits in competitive cross-country skiers during outdoor roller skiing at different competition durations, ranging from the endurance domain to the sprint domain.

**Methods:**

Ten competitive cross-country skiers (6 males; $\dot{\text{V}}$O_2max_ 78 ± 3 and 4 females; $\dot{\text{V}}$O_2max_ 62 ± 3 mL∙kg^−1^∙min^−1^) raced time trials consisting of 1, 2, and 4 laps in a 1.6 km racecourse in a randomized order with 35 min recovery in-between. Oxygen uptake was measured using a wearable metabolic system while oxygen demand was estimated from kinematic data (GPS and IMU) and an athlete-specific model of skiing economy. Skiing economy and $\dot{\text{V}}$O_2max_ was established on a separate test day using six submaximal constant-load trials at different speeds and inclines, and one maximal-effort trial on a roller-skiing treadmill.

**Results:**

Average oxygen demand was 112 ± 8%, 103 ± 7% and 98 ± 7% of $\dot{\text{V}}$O_2max_ during the 1 (3:37 ± 0:20 m:ss), 2 (7:36 ± 0:38 m:ss) and 4 (15:43 ± 1:26 m:ss) lap time trials, respectively, and appeared to follow an inverse relationship with time-trial duration. Average oxygen uptake was unaffected by race length (86 ± 5%, 86 ± 5%, and 86 ± 7% of $\dot{\text{V}}$O_2max_, respectively). Accumulated oxygen deficit at the end of each time trial was 85 ± 13, 106 ± 32 and 158 ± 62 mL∙kg^−1^, while oxygen deficits per work bout was 23 ± 3, 18 ± 3 and 16 ± 3 mL∙kg^−1^ for the 1, 2, and 4-lap time trials, respectively.

**Conclusion:**

Elite cross-country skiers adjust their pacing strategies from attaining relatively small oxygen deficits per work bout in the endurance domain, to larger deficits in the sprint domain. This indicates a shift in strategy from prioritizing stable work-economy and rate-of-recovery in the endurance domain, to maximizing power output in the sprint domain.

## Introduction

In most human-powered time-trial events, the time-trial duration and average speed follow a characteristic, curvilinear relationship (Hill 1925). Specifically, speed initially decreases rapidly as duration increases, before reaching an apparent plateau that can be sustained for a very long time. In some exercise modes, e.g., cycling, power output (*P*) follows a similar relationship with duration. A common method to describe the power-duration relationship, at least in the last few decades (Jones et al. 2010), is an inverse function of duration, or *P* = *CP* + *W*’/*t*. This equation is termed the critical power (CP) model. The asymptote, *CP*, represents a “critical power” output that can be sustained “indefinitely”, while *W*’ represents a finite, constant energy store that is distributed throughout the event.

The CP model has also been applied to exercise modes where power output is fluctuating substantially, termed intermittent exercise modes (Jones and Vanhatalo 2017). During intermittent exercise, it has been shown that *CP* is important in two ways: first, the energy store represented by *W*’ is drained when power output exceeds *CP*, and second, *W*’ is replenished when power output falls below *CP* (Chidnok et al. 2012). From a physiological standpoint, *CP* can be interpreted as a “critical $\dot{\text{V}}$O_2_”, i.e. the highest metabolic rate that can be sustained with aerobic energy provision on the organism-level (Keir et al. 2015; Vanhatalo et al. 2016; Poole et al. 2016). This interpretation is useful in exercise modes where commonly used proxies of exercise intensity, such as power output or speed, do not have a simple link to metabolic energy turnover.

Cross-country skiing is performed in undulating terrain that dictates segments of varying steepness, where the duration of each segment is typically 10–35 s (Losnegard 2019). In uphill segments, skiers choose a pacing strategy with a high energy turnover, typically 100–160% of $\dot{\text{V}}$O_2max_. This high energy turnover is possible due to partial metabolic recovery in downhill segments, as the athletes enter the tucked position (Losnegard 2019).

In a previous study by our group, we measured oxygen uptake, demand, and deficit during a roller-skiing treadmill simulation with duration around 32 min. We found that skiers repeatedly attained relatively small oxygen deficits in-between segments of partial recovery (i.e., downhill segments). However, when summed, the oxygen deficits exceeded the athletes’ maximal accumulated oxygen deficit by a factor 3.8. Assuming that the maximal accumulated oxygen deficit represents a finite anaerobic work capacity, the fact that the overall oxygen deficit exceeded this work capacity almost fourfold indicated a substantial recovery of anaerobic work capacity in the recovery periods. The strategy of repeated but small oxygen deficits is likely beneficial in the endurance domain, where maintaining a stable work economy and a rapid rate-of-recovery from oxygen deficits is essential. However, cross-country skiing competitions range in duration from about 3 min, termed “sprint”, to about 2 h (50 km). It is likely that, as race duration decrease from the endurance domain towards the sprint distance, maintaining work economy and rapid rate-of-recovery become less important, while anaerobic capacity per se becomes more important (Losnegard 2019). Therefore, it would be of interest to study how pacing strategies, i.e., the distribution of effort quantified by instantaneous oxygen demand, of highly skilled cross-country skiers are affected as we move from the endurance domain towards the sprint domain in cross-country skiing. Furthermore, it is of interest to study if average oxygen demand follows the inverse relationship with duration seen in constant-load exercise.

The primary aim of this study was therefore to investigate how oxygen uptake, demand, and deficits are affected by race durations ranging from about 3.5 min (i.e. “sprint”) to > 15 min (well into the endurance domain). We used the CP-model, or rather a “critical $\dot{\text{V}}$O_2_”-model, as a theoretical framework for race-average values of oxygen demand. We hypothesized that average oxygen demand would be inversely related to race duration, similar to constant-load exercise, but that average oxygen uptake would not change with race duration. Second, we hypothesized that differences in speed and oxygen demand between time-trial durations would be more pronounced (i.e., a greater reduction in speed or oxygen demand as duration increased) in the uphill sections of the course. The reasoning behind this hypothesis was that uphill sections have the highest impact on finishing time (Andersson et al. 2010; Sandbakk et al. 2011a; Bolger et al. 2015) and allow higher peak propulsive power outputs (Haugnes et al. 2019). Lastly, we hypothesized that oxygen deficits within individual work bouts would increase as race duration decreased, reflecting the shift in strategy from maintaining work economy and rate-of-recovery in the endurance domain, to maximizing power output in the sprint-distance.

## Methods

### Summary of experimental design

The study consisted of 2 test days separated by 2–7 days. Test day 1 included three individual ski-skating time trials on a roller-skiing track, while test day 2 consisted of laboratory measurements on a roller-skiing treadmill. The three time trials on test day one were conducted on the same track (length 1560 m) with 35 min recovery in-between trials. The length of the time trial was varied by completing either 1, 2, or 4 laps in a randomized order. The athletes wore a portable gas analyser and position-tracking devices with Global Navigation Satellite System (GNSS) receivers and inertial measurement units (IMU) throughout all time trials. The laboratory testing (test day 2) consisted of six different submaximal loads to establish individual models of skiing economy and a ~ 4.5 min maximal-effort test. The maximum effort test was used to measure $\dot{\text{V}}$O_2max_ and calculate maximal accumulated oxygen deficit (MAOD). Finally, oxygen demand during the time trials of test day 1 was estimated by combining propulsive power output (calculated from the position-tracking devices) and the model of skiing economy.

### Subjects

Ten competitive cross-country skiers, six males (age 21.4 ± 5.2 years, body mass 74.8 ± 4.7 kg, $\dot{\text{V}}$O_2max_ 78.0 ± 2.2 ml∙min^−1^∙kg^−1^) and four females (age 24.6 ± 3.8 years, body mass 64.0 ± 7.0 kg, $\dot{\text{V}}$O_2max_ 62.4 ± 3.1 ml∙min^−1^∙kg^−1^), participated in this study after giving written informed consent. Thirteen participants were initially recruited, but three were discarded due to equipment malfunction. Since we did not expect sex-related differences in the study’s outcome variables, we aggregated all participants in one group for the statistical analysis. Inclusion criteria was previous experience in cross-country skiing competitions at a national level and age > 18 years. The study was conducted in line with the rules of the Helsinki-declaration and was approved by the ethical committee of the Norwegian School of Sport Sciences (application 139) and in agreement with the Norwegian Center for Research Data (application 853,738).

### Test day 1: roller-skiing time trials

**Fig. 1:**
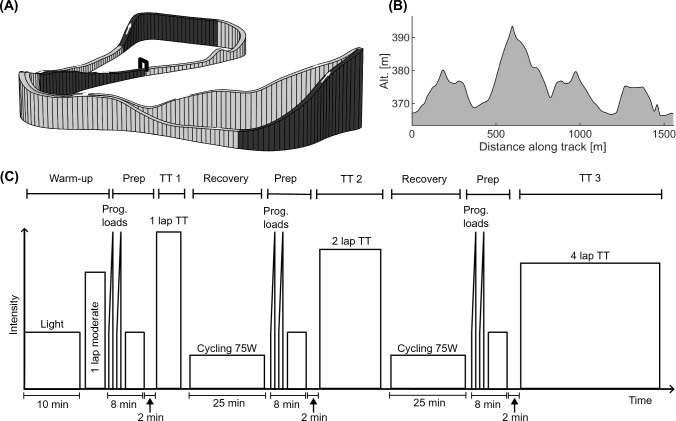
Racetrack topography (**A**) and height profile (**B**). Panel **C** illustrates the experimental protocol for the roller-skiing time trials. The protocol was divided into four phases: warm-up, preparation (Prep), time trials (TT 1, 2 or 3), and recovery. The warm-up phase was only conducted prior to the first TT. It consisted of 10 minutes roller skiing at low intensity followed by a full run of the racecourse at moderate intensity. The preparation-phase lasted 10 minutes and was conducted directly prior to the start of each TT. It consisted of two progressive 30 s loads, finishing at approximately the 1-lap test intensity, followed by 3 minutes of light activity and 3 minutes of standing still. The TT-phase consisted of the time trials, which were either 1, 2, or 4 laps of the racetrack in a randomized order. The recovery-phase consisted of 25 min of ergometer cycling at 75 W

The racecourse (Holmenkollen, Oslo, Norway) was 1560 m long with height difference 28 m and total climb of 65 m per lap (Fig. 1A and B). Upon arrival, the athletes were informed of the order in which to complete the time trials (1-, 2-, and 4-laps). They were instructed to complete each time trial in the shortest time possible, as if it was a cross-country skiing competition. Warm-up prior to the first time trial consisted of 10 minutes of low-intensity roller skiing, followed by a full run of the racecourse at moderate intensity (warm-up phase, Fig. 1C). After the warm-up phase, the athletes completed two progressive 30 s loads finishing at approximately the 1-lap time-trial intensity, followed by 3 minutes of light activity and 2 minutes of standing still (Prep-phase, Fig. 1C). The last 2 minutes of the Prep-phase were used by the experimenter to mount the portable gas analyzer on the athlete, which ensured a similar initial metabolic rate for the succeeding-time trial (TT-phase, Fig. 1C). The three-time trials were separated by 35 min, which consisted of 25 min of ergometer cycling at 75 W (Recovery-phase, Fig. 1C) and the 10 min Prep-phase.

Two position-tracking devices, consisting of a 10 Hz standalone GNSS module and 9-axis inertial measurement unit, were attached to the athlete prior to warm-up. One was positioned in a tight-fitting vest approximately at the level of the third thoracic vertebra, the other was taped laterally on the thigh approximately midway between trochanter and the distal lateral condyle of the femur.

### Test day 2: laboratory tests of skiing economy

Laboratory testing was done in the biomechanics laboratory at the Norwegian School of Sport Sciences (Oslo, Norway). After 15 min of low-intensity roller skiing on the treadmill, the athletes completed six submaximal workloads of 5 min at varying speed and incline, with 2.5 min breaks in-between. During these loads, speed varied from 2.50 to 6.00 m∙s^−1^ for males and 2.25 to 5.40 m∙s^−1^ females, while incline was equal for both sexes (Table 1). This resulted in external work rates between 2.16–2.70 W∙kg^−1^ for males and 1.94–2.43 W∙kg^−1^ for females. The protocol was repeated with speed reductions on a separate day for one of the participants, since exercise intensity of the initial protocol was too high (judged by too high blood lactate and RPE values, and not attaining steady-state $\dot{\text{V}}$O_2_). Pulmonary $\dot{\text{V}}$O_2_ measurements and heart rate data were collected during the workloads, while blood lactate and rate of perceived exertion (RPE, scale from 6–20) were collected at the end of each workload. After a 15 min rest period from the submaximal loads, the participants completed a 1000 m maximal-effort test at 6.0° (males) or 4.5° (females) incline. The athletes could control their speed by moving in front of or behind two laser lines, as previously described by Losnegard et al. (Losnegard et al. 2013). Pulmonary $\dot{\text{V}}$O_2_ was measured throughout the test, and blood lactate and RPE were measured (or reported) at the end of the test.

**Table 1:** Results from submaximal loads (S1-S6) and 1000 m maximal-effort test (Max) during test day 2. All values are presented as average ± standard deviation within each sex since the protocols differed between sexes. Rating of perceived exertion (RPE) was not collected following the 1000 m maximal-effort test, while maximal accumulated oxygen deficit (MAOD) was only defined for the 1000 m maximal-effort trial

	Load	Speed	Incline	$\dot{\text{V}}$O_2_	[La]	RPE	MAOD
		m∙s^−1^	°	mL∙min^−1^∙kg^−1^	mmol	6–20	mL∙kg^−1^
Males (*n* = 6)	S1	6.00	1.07	57 ± 4	2.1 ± 0.9	12.8 ± 0.8	–
	S2	2.50	4.65	50 ± 3	1.6 ± 0.5	12.2 ± 0.4	–
	S3	6.00	1.33	62 ± 4	2.9 ± 1.3	14.1 ± 1.0	–
	S4	4.00	2.12	51 ± 4	1.9 ± 1.0	12.0 ± 1.0	–
	S5	4.00	2.52	54 ± 3	1.9 ± 0.7	13.2 ± 0.8	–
	S6	4.00	2.91	60 ± 3	2.8 ± 1.0	14.7 ± 0.4	–
	Max	3.8 ± 0.2	6.0	78 ± 3	10.3 ± 1.6	–	96 ± 24
Females (*n* = 4)	S1	5.40	1.08	51 ± 4	2.8 ± 1.0	12.3 ± 0.6	–
	S2	2.25	4.66	46 ± 3	2.1 ± 0.4	12.0 ± 2.6	–
	S3	5.40	1.32	53 ± 2	3.4 ± 0.7	13.8 ± 1.6	–
	S4	3.60	2.12	46 ± 2	1.7 ± 0.4	12.0 ± 1.0	–
	S5	3.60	2.52	49 ± 1	2.0 ± 0.4	13.2 ± 1.0	–
	S6	3.60	2.91	52 ± 1	2.7 ± 0.5	14.7 ± 0.6	–
	Max	3.7 ± 0.3	4.5	62 ± 3	9.3 ± 0.9	–	86 ± 18

### Instruments and materials

The participants used the same pair of roller skis on all tests (Swenor skate long, wheel type 2), except for the 1000 m maximal-effort trial on test day 2, where they used a faster wheel type (wheel type 1) due to standardization with respect to previous testing in our laboratory. The skis’ coefficient of rolling resistance (*C*_rr_ = 0.0216) was measured on a flat section of the asphalt roller-skiing track by rolling in the tucked position between four pairs of photo-cells following the method described by Sandbakk et al. (Sandbakk et al. 2011a), but also correcting for air drag using the standard drag equation with drag area 0.35 m^2^ and air density 1.2 kg∙m^−3^. Rolling resistance was measured on the treadmill using a load cell by towing the experimenter at 3 m∙s^−1^ and zero incline, with resulting *C*_rr_ of 0.018 and 0.014 for wheel type 2 and 1, respectively.

On test day 1, the participants were equipped with two Catapult Optimeye S5 position-tracking devices, which consists of a 10 Hz standalone GNSS module and a 100 Hz inertial measurement unit (IMU), including linear accelerometers, gyroscopes and magnetic field sensors. A Cosmed K5 portable gas analyzer in dynamic mixing chamber mode was used to measure $\dot{\text{V}}$O_2_ in all tests, both for test day 1 (field) and test day 2 (laboratory). The gas analyzer was calibrated directly before testing of each participant using a certified calibration gas and a 3 L syringe. Blood lactate was measured using a Biosen C-line analyzer (EKF Diagnostic GmbH, Barleben, Germany).

### Data analysis

Propulsive power (*P*_prop_) was calculated as described by Gløersen et al. (2018), but with some simplifications on the air drag estimates. Specifically, frontal area in the upright position (*A*_0_) was determined using allometric scaling between body mass and frontal area using data from Gløersen et al. (2018). The allometric scaling model was *A*_*0*_ = *k*∙*m*^2/3^, where the proportionality constant *k* = 0.0325 m^2^∙kg^−2/3^ was determined by least squares fitting to the data from Gløersen et al. (2018).

Oxygen demand ($\dot{\text{V}}$O_2_^dem^) was predicted based on the approach presented in Gløersen et al. (2020), which is based on an athlete-specific linear relationship between the cost of transport (*C*, measured in milliliters of oxygen above baseline per meter travelled) and propulsive force (*F*_prop_ = *P*_prop_/*v*):

1 $$\frac{V{O}_{2}^{dem}-V{O}_{2}^{rest}}{v\cdot 60\frac{\text{seconds}}{{\text{minute}}}}={\beta }_{1}\cdot {F}_{\text{prop}}+{\beta }_{0}$$

Here $\dot{\text{V}}$O_2_^rest^ represents baseline metabolic rate, which was set to 5.1 mL∙kg^−1^∙min^−1^, as in (Gløersen et al. 2020). To determine the regression coefficients (*β*_*i*_), the left-hand side in Eq. 1 was evaluated by setting $\dot{\text{V}}$O_2_^dem^ equal to steady-state $\dot{\text{V}}$O_2_ during the submaximal trials and *v* equal to treadmill speed in meters per second. Propulsive power (*F*_prop_) in the right hand side was defined as *m*∙*g*∙(sin *θ* + *C*_rr_∙cos *θ*). The coefficients *β*_*1*_ and *β*_0_ were determined individually for each athlete by ordinary least squares linear regression. Predictions of $\dot{\text{V}}$O_2_^dem^ during the time trials (test day 1) and 1000 m maximal-effort test (test day 2) was achieved by solving Eq. 1 for $\dot{\text{V}}$O_2_^dem^. Oxygen demand during periods where participants were in the tucked position (i.e., not generating propulsion) was set equal to 20 mL∙kg^−1^∙min^−1^. Accumulated oxygen deficit (∑O_2_^def^) was defined as the difference between accumulated oxygen demand and accumulated oxygen uptake during a given time interval. Accumulated oxygen deficit during the 1000 m maximal-effort test was termed “maximal accumulated oxygen deficit” (MAOD).

Oxygen uptake measured by the portable gas analyzer included a time delay, since it was used in dynamic mixing chamber mode. To correct for the delay, we detected when measured $\dot{\text{V}}$O_2_ exceeded initial $\dot{\text{V}}$O_2_ by more than 2.5 mL∙kg^−1^∙min^−1^ during the time trials, which was on average 26 ± 4 s after the start. The $\dot{\text{V}}$O_2_ measurements were shifted in time with this delay for all tests, on both test days.

During the time trials, fractional utilization of VO_2max_ was defined as average $\dot{\text{V}}$O_2_ divided by $\dot{\text{V}}$O_2max_, excluding the first 40 s of each trial from the average to minimize the effect of $\dot{\text{V}}$O_2_ measurement delays. VO_2max_ was defined as the highest 1 min average from the 1000 m maximal-effort test.

### Evaluation of average oxygen demand and skiing speed vs duration

Following the “critical $\dot{\text{V}}$O_2_”-model, individual relationships between time-trial duration and average oxygen demand were established by least squares fitting of average oxygen demand to the inverse of duration, i.e., $\dot{\text{V}}$O_2_^dem^ = *a*_1_∙*t*^−1^ + *b*_1_, where *a*_1_ and *b*_1_ are model coefficients and *t* is duration. The same relationships were also established for average oxygen demand excluding tucked position (i.e., $\dot{\text{V}}$O_2_^dem, excl. tuck^ = *a*_2_∙*t*^−1^ + *b*_2_), and average speed (i.e., *v* = *a*_3_∙*t*^−1^ + *b*_3_). Agreement between modelled and measured oxygen demand was assessed graphically (Fig. 3).

### Statistical analysis

Effects of time-trial length on speed, oxygen demand, oxygen uptake, percentage aerobic contribution, accumulated oxygen deficit, and average accumulated oxygen deficit per work bout (separated by at least 5 s of tucked position) was assessed using repeated measures ANOVA with time-trial length as a categorical within-subject factor. Differences between MAOD and accumulated oxygen deficit at the end of each time trial, and between $\dot{\text{V}}$O_2max_ (test day 2) and $\dot{\text{V}}$O_2peak_ from test day 1, was assessed using repeated measures ANOVA with time-trial length and 1000 m performance trial as categorical within-subject factors.

Post-hoc comparisons were conducted using Bonferroni’s correction. Normality was assessed using Kolmogorov–Smirnov’s test, sphericity was assessed using Mauchly’s test, and Greenhouse–Geisser’s correction was used if sphericity was violated. Differences in instantaneous oxygen demand throughout the race course was evaluated using one-way repeated measures ANOVA with 1D statistical parametric mapping (Pataky et al. 2015) using the SPM1D Matlab toolbox, version M.0.4.10. Post-hoc testing on the SPM1D results was not conducted since methods for this are still under development. Level of significance was set to α = 0.05 for all tests. All analysis were performed in Matlab R2022b (The MathWorks, Natick, USA). Data from three of the participants were discarded due to erratic measurements from the portable gas analyzer (most likely caused by saliva affecting the flow turbine) or missing GNSS measurements (receiver out of battery). Hence, the analyses were performed on six males and four females. Results are presented as mean ± standard deviation (SD).

## Results

Results from the submaximal and 1000 m maximal-effort tests (test day 2) are presented in Table 1. The regression model coefficients (Eq. 1) were β_1_ = 3.6 ± 0.6 μL∙kg^−1^∙m^−1^∙N^−1^ and β_0_ = 48 ± 13 μL∙kg^−1^∙m^−1^.

### Time trials

**Fig. 2:**
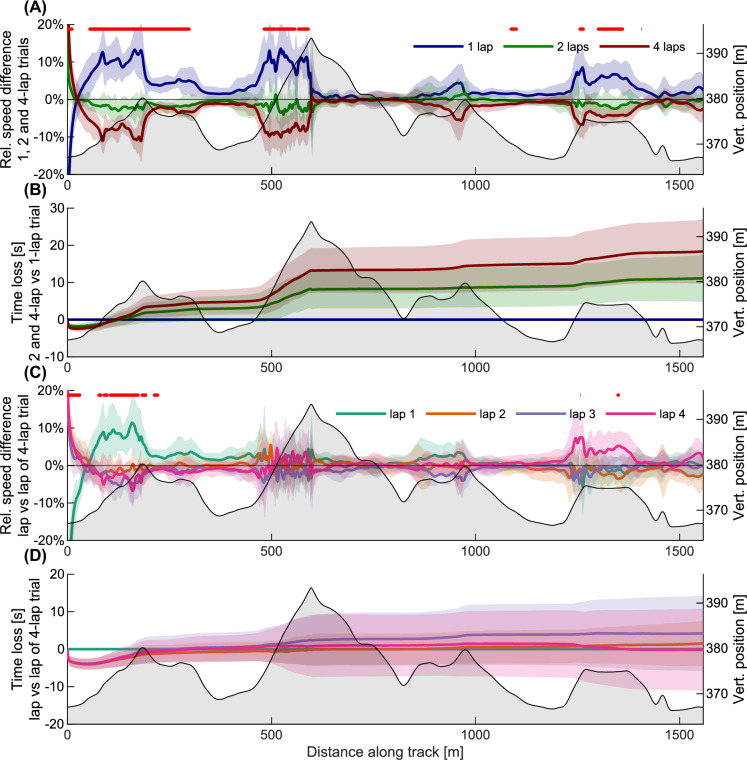
Pacing strategies at different competition lengths. The horizontal axis on all plots show distance along the track, with the altitude profile in light gray in the background. **A** Relative speed difference from the average of all competition lengths. The intermittent red line indicates a significant difference (SPM1D ANOVA, *α* = 0.05) between the different conditions (i.e., race lengths). **B** Time gain or loss compared to the 1-lap condition. **C** Speed difference between laps during the 4-lap time trial, relative to average speed during the 4-lap condition. The intermittent red line indicates a significant difference (SPM1D ANOVA, *α* = 0.05) between the different conditions (i.e., lap number during 4-lap trial). **D** Time gain or loss during the laps on the 4-lap time trial compared to the first lap of the 4-lap time trial

The relative speed differences and accumulated time losses between the 1-, 2-, and 4-lap conditions, and between the different laps of the 4-lap condition, are shown in Fig. 2. Average speed and duration for the different conditions are presented in Table 2. On average, the 1-lap condition was 5.4 ± 2.9% faster than the 2-lap condition (*p* < 0.001, post-hoc *t* test) and the 2-lap condition was 3.1 ± 1.7% faster than the 4-lap condition (*p* < 0.001, post-hoc *t* test). The speed differences were more pronounced in uphill segments, and specifically at the major climb between 450 and 600 m (Fig. 2A, B) (*p* < 0.05, repeated measures ANOVA using SPM1D). The comparison between laps for the 4-lap condition (Fig. 2C, D) showed that skiers had a higher speed during the initial phase (first ⁓500 m, *p* < 0.05, repeated measures ANOVA using SPM1D) of the race, followed by a similar pacing profile until the last uphill, where they skied faster on the last lap (the last ⁓300 m of the race), although this was mostly not significant at the α-level of 0.05 (ANOVA using SPM1D, Fig. 2C).

**Table 2:** Time trial results

Laps	Duration	Speed	$\dot{\text{V}}$O_2_^dem^		$\dot{\text{V}}$O_2_	$\dot{\text{V}}$O_2peak_	Fract. util	∑O_2_^def^	Aerob. contrib
	s	m∙s^−1^	mL∙kg–^1^∙min^−1^		mL∙kg^−1^∙min^−1^	mL∙kg–^1^∙min^−1^	%	mL∙kg^−1^	%
1	217 ± 20	7.2 ± 0.6	81 ± 9		62 ± 8	65 ± 9^†^	86 ± 6	88 ± 13	70 ± 3
2	456 ± 40	6.9 ± 0.6^*^	74 ± 8^*^		62 ± 7	68 ± 8^*^	86 ± 6	113 ± 30	80 ± 5^*^
4	943 ± 91	6.7 ± 0.6^*^	70 ± 8^*^		61 ± 8	70 ± 9	86 ± 7	164 ± 62^†^	85 ± 5^*^

^*^Significant change from the row above, *p* < 0.05. ^†^Significant change from 1000 m maximal-effort trial (test day 2), *p* < 0.05. $\dot{V}$*O*_*2*_^*dem*^ oxygen demand, *Fract. util.* fractional utilization of $\dot{\text{V}}$O_2max_, *∑O*_*2*_^*def*^ accumulated oxygen deficit, *Aerob. contrib.* fraction of energy contribution from aerobic sources (accumulated oxygen uptake divided by accumulated oxygen demand). $\dot{\text{V}}$O_2_ measurements prior to 40 s excluded from the average for $\dot{\text{V}}$O_2_ and fractional utilization of $\dot{\text{V}}$O_2max_

**Fig. 3:**
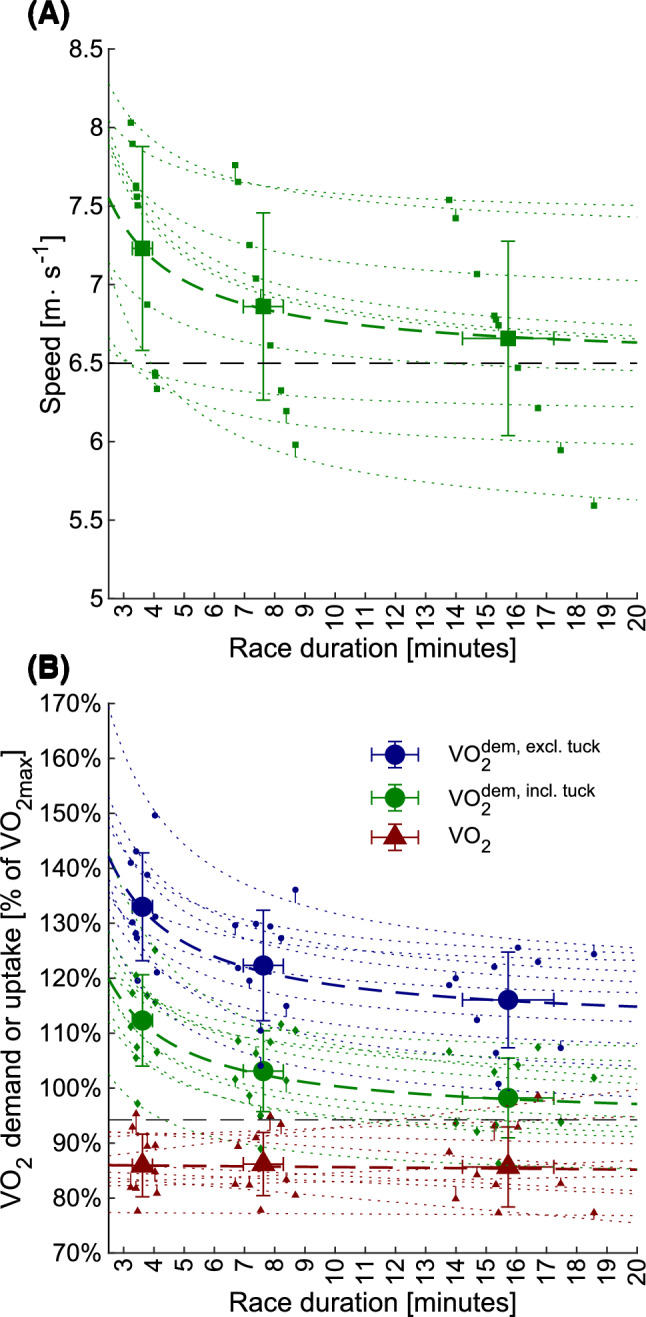
**A** Speed vs time-trial duration. Error bars indicate between-athlete standard deviation. Individual datapoints and model fits are displayed as small squares and dotted lines, respectively. Dashed black line indicates the horizontal asymptote (“critical speed”). **B** Oxygen demand or uptake normalized to $\dot{\text{V}}$O_2max_ vs race duration. Individual datapoints and model fits are displayed with small markers and dotted lines, respectively. Circles: oxygen demand excluding periods in tucked position; diamonds: oxygen demand including periods in tucked position; triangles: average oxygen uptake (excluding the first 40 s of each time trial)

Average oxygen demand and speed were affected by time-trial length (*p* < 0.001, *F* = 79 and *F* = 50, respectively, repeated measures ANOVA) and appeared to follow an inverse relationship with time-trial duration (Fig. 3, Table 2). Average oxygen uptake (excluding the initial 40 s) was unaffected by time-trial length (*p* = 0.86, *F* = 0.16, repeated measures ANOVA, Table 2). The fraction of energy release from aerobic sources (accumulated oxygen uptake / accumulated oxygen demand) was different between conditions (*p* < 0.001, *F* = 142, repeated measures ANOVA) and increased with time-trial length (Table 2). Accumulated oxygen deficit at the end of each time trial was different between conditions (*p* < 0.002, *F* = 14.0, repeated measures ANOVA, Table 2). Post-hoc *t* tests indicated that accumulated oxygen deficits during the 4-lap trial exceeded MAOD from the 1000 m maximal-effort trial (test day 2) by 84 ± 75% (*p* = 0.006), while accumulated oxygen deficits from the 1- and 2-lap time trials were not different from MAOD (*p* = 0.10–0.55). Peak oxygen uptake was also different between conditions (*p* < 0.001, *F* = 7.6, repeated measures ANOVA, Table 2). Post-hoc *t* tests indicated that $\dot{\text{V}}$O_2peak_ during the 1-lap condition was less than $\dot{\text{V}}$O_2max_ during the 1000 m maximal-effort trial (*p* < 0.001), while $\dot{\text{V}}$O_2peak_ during the 2- and 4-lap conditions were not different from $\dot{\text{V}}$O_2max_ (*p* = 0.05–0.31).

The average oxygen deficit incurred per bout of active skiing (i.e., separated by at least 5 s of tucked-position) was affected by time-trial length (*p* < 0.001, *F* = 88, repeated measures ANOVA). Post-hoc *t* tests showed that average oxygen deficit per bout was reduced from 23.1 ± 2.5 to 17.6 ± 2.5 mL∙kg^−1^ between the 1- and 2- lap conditions (*p* < 0.001), and from 17.6 ± 2.5 to 15.8 ± 3.2 mL∙kg^−1^ between the 2- to 4- lap conditions (*p* = 0.02).

**Fig. 4:**
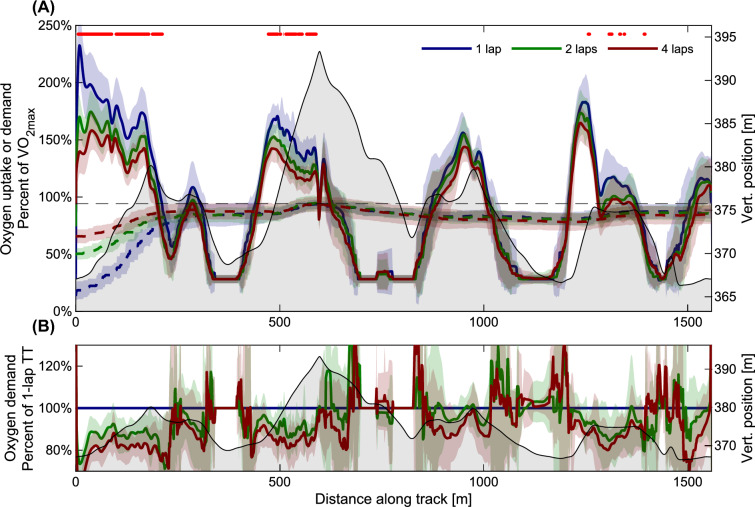
**A** Oxygen demand and uptake normalized to $\dot{\text{V}}$O_2max_ as a function of distance along the track. Solid lines: oxygen demand, dashed lines: oxygen uptake. The lines indicate values averaged over all participants, shaded areas indicate ± one standard deviation. Red line in the top part indicates a significant difference between conditions (time-trial length) at that point in the track (SPM1D ANOVA, *α* = 0.05). **B** Oxygen demand relative to the 1-lap time trial. Color coding and error bars follow the convention in panel A. The altitude profile of the track is shown in light gray in both panels, right side y-axis

**Fig. 5:**
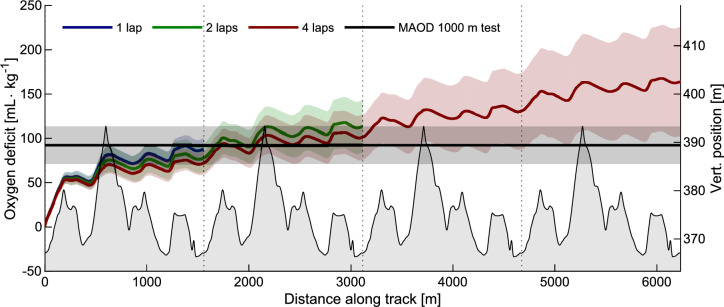
Oxygen deficits as a function of distance along the track. Maximal accumulated deficit (MAOD) from the 1000 m maximal-effort test on test day two in shown as a black line. Shaded areas indicate one standard deviation

The participants’ instantaneous $\dot{\text{V}}$O_2_^dem^ and $\dot{\text{V}}$O_2_ uptake as a function of racecourse position is shown in Fig. 4. During uphill segments, participants raced at supra-maximal oxygen demands, attaining peak values of two times their $\dot{\text{V}}$O_2max_ in the 1-lap condition. Instantaneous $\dot{\text{V}}$O_2_^dem^ was different between conditions (1, 2 or 4-laps) in the first and second uphill segments, and during the last flat segment (repeated measures ANOVA using SPM1D, red horizontal line in Fig. 4A). The gradual accumulation of oxygen deficits throughout each time trial is shown in Fig. 5.

## Discussion

This study is the first to measure oxygen uptake and estimate oxygen demand continuously throughout time trials of durations ranging from sprint (~ 3.5 min), to distance (~ 15.8 min) during roller-skiing in an international racecourse. In line with our first hypotheses, we found that average oxygen demand appeared to follow an inverse relationship with race duration, while average oxygen uptake was unaffected by duration. Partially in line with our second hypothesis, we found significant differences in instantaneous oxygen demand in the two main uphill sections of the course. However, there were also significant differences in oxygen demand in the last flat section of the course. We also found support for our third hypothesis since oxygen deficits within individual work bouts decrease as race duration increased.

### Oxygen uptake and demand during roller-skiing time trials

Average oxygen demand during the 1-lap trial was 112 ± 8% of $\dot{\text{V}}$O_2max_, which is in fair agreement with the findings by Losnegard et al. (111–113% of $\dot{\text{V}}$O_2max_, duration 172 s) (Losnegard et al. 2012), but higher than the findings of Andersson et al. (101–104% of $\dot{\text{V}}$O_2max_, duration 230 s) (Andersson et al. 2017). Sandbakk et al. (Sandbakk et al. 2011a) estimated work rate to be 160% of peak aerobic power during a relatively long uphill segment (50 s), which is similar to the longest uphill segment in the current study. Hence, the values observed in the current study appear to agree with previous estimates of both time-trial averages and course-section averages.

The finding that oxygen uptake reaches $\dot{\text{V}}$O_2max_ during the 2- and 4-laps time trials differs from the findings of Welde et al. (Welde et al. 2003) and Gløersen et al. (Gløersen et al. 2020), who both measured oxygen uptake throughout distance skiing time trials (Welde using a portable gas analyzer while skiing on snow, Gløersen using a laboratory analyzer during a treadmill simulation). Both these studies reported that $\dot{\text{V}}$O_2max_, as measured during a separate maximal-effort trial, was not reached during the skiing time trials. Both Welde and Gløersen used time trials with longer finishing time than the 4-laps time trial of the current study, which might explain the discrepancy from the current study. Hence, this indicates that cross-country skiers might attain oxygen uptakes equal to $\dot{\text{V}}$O_2max_ during skiing time trials with duration longer than about 4 min but shorter than about 20 min. This is true even if the exercise mode is of intermittent nature.

### Energy system contributions

For the 1-lap time trial, our results indicated an anaerobic energy contribution of 30% at a race duration of 217 s. This is a larger contribution than expected from laboratory-based studies on sprint skiing, where Losnegard et al. (Losnegard et al. 2012) found a 26% anaerobic contribution at 170 s race duration for ski skating, and Andersson et al. (Andersson et al. 2017) found a 18% anaerobic contribution at 232 s duration. Anaerobic contribution for the 1-lap and 2-lap time trials were also higher than expected based on the review across different sports by Gastin et al. (Gastin 2001), which indicated a 24% and 10% anaerobic contribution for the average 1-lap and 2-lap durations in the current study, respectively. The higher-than-expected anaerobic contributions might be explained by differences between treadmill simulations and time trials on a competition track, which included both steep uphill segments (average 9° incline for the main uphill section), downhill segments where the tucked position was used, and turns. It has been shown that both peak propulsive power (Haugnes et al. 2019) and the ability to accumulate oxygen deficits (Karlsson et al. 2018) increase with incline (or decrease with speed) in ski skating. This might indicate that courses with steep uphill sections facilitate larger anaerobic contributions, since both the capacity to attain oxygen deficits, and the rate at which they can be attained, is high. It is difficult to conclude how the presence of downhill segments, where no propulsion is generated, affects the ability to incur and replenish oxygen deficits, but the results of the present study might suggest that such brief periods of recovery can increase the overall anaerobic contribution to energy release, at least when defined as in the current study.

### Practical applications

The findings in the current study indicate that cross-country skiing sprints put a greater demand on anaerobic capacity and power compared to more constant-load sports of similar duration, which should be reflected in the training. Active muscle mass is likely to increase anaerobic capacity (Olesen 1992), and (Losnegard and Hallén 2014) found that sprint skiers had greater body mass and BMI than their distance skier counterparts. However, interventions studies on resistance training (Losnegard et al. 2011; Skattebo et al. 2016) show a trivial effect on skiing performance. Furthermore, Talsnes et al. (2024) did not find a relationship between anaerobic power, measured as a 30 s double poling ergometer test, and sprint performance. However, they did find a relationship between peak skiing speed (both uphill and flat) and sprint performance. Taken together, this can indicate that specificity in the training of anaerobic power or capacity is key to utilize improved anaerobic abilities in sprint skiing. This is accentuated by the fact that cross-country skiing includes frequent shifts between movement patterns, which are used as a gearing system (Nilsson et al. 2004; Sollie et al. 2021). Finally, in addition to an initial time-trial, cross-country skiing sprint competitions consists of three head-to-head heats separated by at least 20 min. The current study did not include such head-to-head heats. It has been shown that maximal aerobic power become more important towards the final heats (Talsnes et al. 2024), and aerobic fitness is undoubtably also a key performance factor for sprint skiers.

The highly dynamic oxygen demands in Fig. 4 show that skiers rarely compete close to their “threshold-intensity”, i.e. their critical $\dot{\text{V}}$O_2_. This is true also for competition durations around 30 min (Gløersen et al. 2020). The principle of training specificity suggests that exercise over the range of speeds and intensities used in competition is beneficial. This does not mean that threshold-intensity training is irrelevant for cross-country skiers, however, it does give some support to the tradition of “polarized” training, i.e. mixing low- (below threshold) and high-intensity (above threshold) training, used by many elite cross-country skiers (K. S. Seiler et al. 2006; Losnegard et al. 2013; Sandbakk and Holmberg 2017). Haugnes et al. (2019) demonstrated that during low- and moderate intensity exercise, the fraction of power output to peak attainable power output is higher in flat terrain compared to uphill. Hence, training at competition-relevant intensities comes at a smaller cost in flat terrain compared to uphill. Therefore, they suggested that skiers should focus their high-intensity training in uphill terrain, to maximize training at relevant intensity in this terrain. While we support this rational, the findings of the current study indicate that moderate and high-intensity training in undulating terrain (mimicking competitions courses, e.g., roller-skiing tracks) can be beneficial. Oxygen demand in the uphill segments in the current study was typically 150% of $\dot{\text{V}}$O_2max_, which is substantially higher than what is attainable during interval training with interval durations > 4 min.

Lastly, Holsbrekken et al. (2023) showed that elite skiers had a longer time to exhaustion than recreational skiers during an intermittent exercise protocol with average intensity equal to their maximal aerobic power, indicating that elite skiers have an increased ability to recover from repeated supra-maximal efforts. Stöggl and Björklund (2017) found that extensive high-intensity interval training improved performance and heart rate recovery in an intermittent exercise test, indicating that the ability to recovery rapidly is trainable through high-intensity interval training at 90–95% of maximal heart rate (although their test had substantially lower work/recovery fraction than a typical cross-country skiing competition). Since recovery from supra-maximal efforts is an aerobic process, it seems likely that this ability can be improved through high-intensity aerobic training which increases oxygen transport to the working muscles. How different training practices affect peripheral factors in the muscles, which can accelerate recovery when oxygen supply is plentiful, is less clear.

In summary, cross-country sprint skiers should aim for specificity in their anaerobic training, to ensure that improvements in anaerobic abilities can be leveraged during skiing. Sprint skiers should also prioritize aerobic training, since aerobic energy release is dominant (70% of total energy release for the sprint distance in the current study). For distance skiers, aerobic power is clearly most important, since this affects both the ability to maintain a high average energy turnover over time, but also (most likely) the ability to recover rapidly from supra-maximal efforts.

### Linking race duration, pacing strategy and bioenergetics

It has been suggested by both ourselves (Losnegard et al. 2015; Gløersen et al. 2020) and others (Björklund et al. 2011; Sandbakk et al. 2011b) that rate of recovery from oxygen deficits might be an important performance criterion in distance cross-country skiing. In (Gløersen et al. 2020), the rationale for this argument was observations of repeated oxygen deficits that were individually of modest magnitude (average 14% of MAOD), but in sum exceeded MAOD by a factor 3.8. We speculated that the small, repeated, oxygen deficits could be sustained because of the relatively fast recovery kinetics of alactic energy sources. These energy sources comprise approximately 30% of MAOD (Gastin 1994) and have a recovery half-time of about 20 s (Margaria et al. 1969; McCully et al. 1994), which is fast enough to allow partial recovery of anaerobic work capacity in downhill sections. If larger oxygen deficits were attained repeatedly, this would be associated with substantial anaerobic glycolysis and glycogenolysis, which might reduce work economy (Jones et al. 2011; Hoff et al. 2016) and the rate of recovery of alactic energy stores (McCully et al. 1994). An intervention study on cross-country skiers’ pacing strategies, where the participants completed a race using both their preferred strategy and a conservative start strategy, showed that the conservative start resulted in shorter finishing time (Losnegard et al. 2022). Maintaining work economy and rate of recovery is of major importance during distance skiing (e.g. about 30 min), but become less important at sprint duration (about 3 min). Rather, the focus shifts towards maximizing speed throughout the course. This shift in strategy finds some support in our results, as the average oxygen deficit attained in each work period gradually decrease as time-trial length increases.

### Effects of topography and race duration on oxygen demand

Although the results of the current study show that average oxygen demand followed an inverse relationship with duration, Losnegard (8) have argued that instantaneous oxygen demand in cross-country skiing is mostly influenced by course topography. This argument was based on similar oxygen demands (160% of $\dot{\text{V}}$O_2peak_) in uphill segments of both sprint and distance skiing. In this study, we found that increased time-trial duration led to a relatively consistent reduction in oxygen demand throughout the course, except for periods where the skier was in the tucked position (which, admittedly, is equal by model assumption). Differences in instantaneous oxygen demand was greatest in uphill segments, while the relative differences between conditions were reasonably constant throughout the course (Fig. 4). Although the differences in oxygen demand incurred by varying race duration were consistent, they were modest in magnitude compared to the substantial variations in instantaneous oxygen demand throughout the course (i.e. 140–200% of $\dot{\text{V}}$O_2peak_ in uphill sections vs 90–120% of $\dot{\text{V}}$O_2peak_ in flat sections, respectively).

### Methodological considerations

The model for oxygen demand assumes an individual-specific and time-constant linear relationship between oxygen demand, propulsive power and skiing speed. The model has been evaluated at submaximal efforts and demonstrated acceptable validity, however, extrapolation to supra-maximal demands is challenging to validate. Particularly, a gradual loss of energy conversions efficiency is believed to occur at work rates exceeding the “critical $\dot{\text{V}}$O_2_”. The extent to which the continuous accumulation of oxygen deficits with time, particularly at the 4-laps condition, can be attributed to such a loss of efficiency, or other aspects of the model, is hard to assess. Recent advances within modelling of $\dot{\text{V}}$O_2_-kinetics include time-dependent energy conversion efficiencies (Gløersen et al. 2022). Further development of this methodology might elucidate this aspect.

The portable gas analyzer used in this study has been found to systematically overestimate $\dot{\text{V}}$O_2_ when used in mixing chamber, both by others (Perez-Suarez et al. 2018) and ourselves during pilot testing prior to this. This systematic overestimation is of little concern for the current study, where the aim was to compare differences within each participant, relative to their own $\dot{\text{V}}$O_2max_. The mixing chamber configuration was used due to the superior reliability it has demonstrated at high metabolic rates (Winkert et al. 2020). This, however, implied a need to correct for measurement delays of $\dot{\text{V}}$O_2_, as explained in the methods section. The choice of using mixing chamber mode should be considered when interpreting the oxygen deficit results.

## Conclusion

This study showed that average oxygen demand in cross-country skiing, which is an intermittent endurance sport, appeared to follow an inverse relationship with time-trial duration. This agrees with expectations from constant-load exercise. The athletes’ pacing strategies shifted from attaining relatively small oxygen deficits per work bout in the distance-domain (on average 17% of MAOD at a duration of 16 min) to larger deficits in the sprint-domain (on average 25% of MAOD at a duration of 3.6 min). This can indicate a shift in strategy from maintaining stable work economy and rapid rate-of-recovery in the distance-domain, to maximizing power output in the sprint-domain. Lastly, the anaerobic contribution to total energy release was higher than reported for most other sports for the 1- and 2-lap conditions (3.6 and 7.6 min).

## Data Availability

The datasets generated and analysed during the current study are available from the corresponding author on reasonable request.

## Abbreviations

ANOVA: Analysis of Variance
*C*: Cost of Transport
CP: Critical Power
*C*_rr_: Coefficient of rolling resistance
*F*_prop_: Propulsive force
GNSS: Global Navigation Satellite System
IMU: Inertial Measurement Unit
MAOD: Maximal Accumulated Oxygen Deficit
*P*_prop_: Propulsive power
rANOVA: Repeated Measures Analysis of Variance
RPE: Rate of Perceived Exertion
$\dot{\text{V}}$O_2_: Rate of oxygen consumption
$\dot{\text{V}}$O_2_^dem^: Oxygen demand
